# Age, Gender, and Ethnicity of Counsellor Trainees and Corresponding Counselling Self-Efficacy: Research Findings and Implications for Counsellor Educators

**DOI:** 10.1007/s10447-012-9175-3

**Published:** 2012-10-20

**Authors:** Sarah Lam, Susan Tracz, Christopher Lucey

**Affiliations:** 1 Department of Counsellor Education and Rehabilitation, California State University, Fresno, 5005 North Maple Avenue, M/S ED 3, Fresno, CA USA; 2 Educational Research and Administration Department, California State University, Fresno, 5005 North Maple Avenue, M/S ED 303, Fresno, CA USA

**Keywords:** Multicultural counselling, Diversity status, Ethnicity, Age, Gender, Counselling self-efficacy, Counsellor education, Counsellor trainees

## Abstract

This study explored the counselling self-efficacy of students in a counsellor education programme, in regard to age, gender, and ethnicity characteristics. To assess counselling self-efficacy, the Counselling Self-Estimate Inventory (COSE) of Larson *et al.* (Counsellor Education & Supervision 41: 120–130, 1992) was administered at the end of a semester to counselling students engaged in different stages of a counsellor training program. No significant differences were found in regard to gender and age-group categories, but significant differences were found among ethnic groups. It was found that Asian and White students generally had similar and also lower counselling self-efficacy means than the other ethnic groups in the sample in regard to several counselling-specific categories. Implications for counsellor educators in training counselling students of diverse characteristics are discussed.

## Introduction

Since the 1960’s, the multicultural movement in counselling has raised research interest in and awareness of the counselling needs of culturally different clients (Heppner *et al.* 2009). Multicultural counselling addresses diverse populations and the influences of culture on the counselling relationship, which includes “the unique cultural background of [the] mental health professionals” (Gerstein *et al.* 2009, p. 22). Gelso (2010) suggests that the diversity status of the counsellor is “significantly under-addressed” in the discourse about multicultural counselling (p. 143). Even when the diversity status of the counsellor is addressed, oftentimes the focus is the impact on therapeutic alliances and treatment outcomes (Wintersteen *et al.* 2005) rather than on the counsellor’s professional development.

To identify the interconnectedness between counsellor diversity status and professional development, this study explored the relationship between counselling students’ age, gender, and ethnicity and their counselling self-efficacy. Larson (1998) postulates counsellors’ demographic factors and counselling self-efficacy as being internal factors involved in counsellor development. Counselling self-efficacy refers to the belief that one has the “capabilities to effectively counsel a client” (Larson *et al.* 1992, p. 120). Counselling self-efficacy regulates trainees’ functioning in making counselling responses (Larson 1998), and consequently plays an important role in trainees’ performances (Jaafar *et al.* 2009; Larson *et al.* 1992; Lent *et al.* 2006). According to the social cognitive theory of Bandura (1995), people’s self-efficacy develops from experiences of mastery, observation of others’ mastery, social affirmation of their mastery, and their perception of physiological or emotional states during the experience. Cultural contexts, which include trainees’ specific age, gender and ethnicity, may impact these four sources of self-efficacy (Klassen 2004) and shape the development of trainees’ sense of competence (Pope-Davis *et al.* 1994).

People from a specific age, gender, and ethnic grouping have typically dominated the counselling profession in the U.S. Riemersma (2010) conducted a demographic survey of Marriage and Family Therapists (MFT) in California with results showing that above 70 % of MFT’s were female, had an average age of 56.4 years, and that above 90 % were White. The same percentage of female dominance has been reported in the number of undergraduate majors in counselling psychology in the U.S. (Carnevale *et al.* 2011). To diversify the cultural backgrounds of counsellors, the Council for Accreditation of Counselling and Related Educational Programs (CACREP) (2009) has required that counsellor education programmes make “systematic efforts to attract, enrol, and retain a diverse group of students and to create and support an inclusive learning community” (p. 4). To accomplish this mission, counsellor educators are encouraged to consider the potential impact of diversity status on their students’ professional development. Diversity includes differences in “races, economic backgrounds, ages, ethnic backgrounds, genders, sexual orientations, and physical and mental abilities” (CACREP 2009, p. 108). The scope of the present study, however, is limited to age, gender, and ethnicity factors.

Trainees’ age reflects both developmental stages and generational affiliation. Using Erikson’s ‘Identity Development Theory’, Evans *et al.* (2010) outlined the main concerns of college students in different age groups as follows: intimate relationship for young adults, professional establishment for the middle-aged, and significant changes in thought patterns for older adults (p. 51). Counsellor trainees’ counselling self-efficacy may to some extent reflect their relative success in accomplishing such developmental tasks. Shallcross (2009) alerts us to the presence of generational identities that may affect one’s sense of self: ‘Baby Boomers’ (50 years old and above) seem to have a strong sense of group identity; ‘Generation Xers’ (30–50 years old) a strong sense of individualism and autonomy; and ‘Generation Yers’ (10–30 years old) value both their identity as individuals and as members of a group.

Differences in the sense of self may affect the development and/or expression of self-efficacy. Furthermore, people’s mastery of certain knowledge and skills may change as they age. Their perception of generation-related abilities may be affected by how much they value the skills and knowledge they have mastered for current job expectations. For example, one’s cognitive self-efficacy may change across the lifespan due to the changing nature of the tasks one performs and the subsequent demands on the domain of cognitive functioning (Berry and West 1993). Similarly, age differences significantly affect how people perceive their efficacy in memory and specific knowledge domains (Marquié and Huet 2000). Understanding whether age differences have a significant impact on trainees’ counselling self-efficacy could increase counsellor educators’ sensitivity to trainees’ individual stages in life and their generational beliefs and values.

Besides the age factor, Wester and Vogel (2002) urge counsellor educators to consider the impact of gender role conflict (GRC) on counselling self-efficacy, particularly that of male counsellor trainees. They argue that males typically are socialized to be more concerned about independence and more restricted in emotional expression than females (p. 370). Males may struggle with expressing feelings, relating to supervisors, openly sharing reflections on the training process, and using relatively feminine styles of counselling. However, Betz and Hackett (1981) found that male college students showed consistent self-efficacy in both traditionally female and male occupations (p. 408). Even if trainees are not affected by their personal gender role socialization, their clients’ perceptions of counselling quality based on gender role orientation and sex (Beckenbach *et al.* 2009) may impact trainees’ development of counselling self-efficacy. Further research on the relationship between gender and counselling self-efficacy is warranted.

Race/ethnicity is another major component of counsellor trainees’ diversity status. The impact of ethnicity on professional efficacy has been indicated in other settings. For example, Klassen (2004) concluded from reviewing 20 studies on self-efficacy that non-Western cultural groups showed a lower level of self-efficacy. The subjects or domains of these studies included managers or employees in business sectors, school children, and college students, and the task or domain for self-efficacy included decision making, social and/or academic situations, business tasks, and self-regulatory capacity.

The potential impact of race/ethnicity on professional self-efficacy may arise from the process of racial/ethnic identity development. Unique manifestations of different stages of racial/ethnic identity development have been detailed for Whites (Helms 1993; Rowe *et al.* 1994); Latinos (Ferdman and Gallegos 2001); Asians (Kim 2001); American Indians (Horse 2001); Blacks (Cross 1991); and Mixed-race individuals (Renn 2003). One manifestation that appears consistently across ethnic groups is a change in self-concept with regard to how they compare themselves to people from the dominant group prior to identity exploration, during identity exploration and after identity achievement (Evans *et al.* 2010). This process is also intertwined with acculturation; i.e., the degree of one’s adoption of beliefs, values, and behaviours of the dominant culture (Evans *et al.*, p. 276). For example, Kim and Omizo (2005) found that Asian American college students who adhered to Asian values tended to have stronger collective self-efficacy, while Asians who adhered to European American values tended to have stronger individual self-efficacy. Also, LaFromboise *et al.* (1993) emphasized that the level of competence in understanding, functioning, behaving, and communicating in two different cultures in the development of bicultural identity varied as a function of the level of integration with mainstream identity.

One question is whether ethnicity impacts the professional self-efficacy of counsellor trainees? Like other college students, counselling students wrestle with their own process of racial/ethnic identity development. At the same time, counselling students face the challenges of being trained in a profession dominated by Whites and being asked to play roles and demonstrate communication styles developed from Western belief systems that may not translate readily to their own cultural backgrounds. For example, counsellor education programmes typically require trainees to demonstrate core conditions in establishing helping relationships, one of which is empathy (Crutchfield *et al.* 2000; Pearson 1999; Rogers 2007). Empathy is the “ability and effort to place oneself symbolically in the position of the client and understand the client’s world” and also “the ability and skill to communicate and demonstrate it” (Chung and Bemak 2002, p. 154). To demonstrate that awareness and understanding, counselling students are expected to openly address feelings in verbal communication. Similarly, another core condition called “confrontation” is to challenge clients on discrepancies in their expressions. Trainees from some cultures may not value the open expression of feelings or condone the behaviour of pinpointing others’ feelings (Wohl 1982, pp. 219–220). Similarly, Zane *et al.* (1991) argue that Asian trainees who have been socialized to be non-assertive and quiet may struggle over confronting clients. Their cultural beliefs in counsellors as experts with authority over clients and the value of personal sacrifice for the collective system are in conflict with counselling values of egalitarianism and individualism. Consequently, Asian students may find it difficult to demonstrate “confrontation, transference interpretation or even reflection of feeling” (Wohl 1982, p. 220). Furthermore, developing self-efficacy involves a sense of personal agency; i.e., the sense of one’s impact on the outcome of one’s action (Mutchler and Anderson 2010). This approach to self-perception may be unfamiliar to Asians who are not used to “looking at oneself as an actor…and…the immediate ‘cause’ of one’s own living” (Wohl 1982, p. 219).

Available studies on the diversity status of counsellor trainees and their self-efficacy tend to focus on self-perceived multicultural competence with mixed findings being reported. For example, using the Multicultural Counselling Inventory (MCI) to measure multicultural self-efficacy among doctoral interns at a university counselling centre, Pope-Davis *et al.* (1994) failed to find significant relationships between the demographics of interns and their multicultural counselling self-efficacy. However, a later study by Pope-Davis *et al.* (1995) involving graduate students in Psychology found a significant difference on multicultural self-efficacy by ethnicity, with ethnic students showing a higher sense of competence than their White peers, while age and gender did not yield significant results. Pope-Davis *et al.* concluded that the different experiences of Whites and persons of colour might account for the difference in their multicultural self-efficacy. Pope-Davis *et al.* affirm the long-held notion that trainees’ personal experiences and beliefs have impact on the development of their sense of competence. Since “efficacy beliefs…remain important factors in the motivational functioning of people from individualist and collectivist cultural groups” (Klassen 2004, p. 228), more research on the counselling self-efficacy of students from different ethnic groups is needed.

Despite some studies on the relationship between trainees’ diversity status and their professional self-efficacy, research done specifically on the relationship between trainees’ age, gender, and ethnicity and their overall counselling self-efficacy is still lacking. A search conducted on PsycINFO on February 28, 2011 using phrases that included counselling “self-efficacy” and “age,” “gender,” “cultural diversity,” or “ethnicity” failed to generate any results. Exploring age, gender, and ethnicity of counsellor trainees and their corresponding counselling self-efficacy may provide a general sense of how diversity status impacts trainees’ counselling self-efficacy and sensitize counsellor educators to consider different approaches to cultivating trainees’ professional efficacy within cultural contexts. In conducting such an exploration, this study attempts to find answers to the following question:

Are there significant differences in counselling self-efficacy among counsellor education students of different age groups, genders, and ethnic backgrounds?

## Method

### Participants

This cross-sectional study was conducted in a U.S. public university in a medium sized city near the West coast. Participants were graduate students in an ethnically diverse counselling education programme, embedded in an ethnically diverse university campus and local community. At programme level, the full-time faculty was made up of White males (3), White females (2), Hispanic males (2), Asian females (2), and a Black female (1). About 300 counselling students were enrolled during the semester when this study took place, which included American Indian (1 %), Asian (9 %), Biracial (1 %), Black (5 %), White (30 %), and Latino (52 %) students. The Asian students category (*N* = 26) included Asian Indians (*N* = 5, 19 %), Chinese (*N* = 4, 15 %), Hmongs [from Laos origins] (*N* = 13, 50 %), Japanese (*N* = 2, 8 %), and Koreans (*N* = 2, 8 %). The Latino students came from different regions of Mexico, Cuba, Puerto Rico, and South America. Black students were African Americans who were born and raised in the U.S.

Most counselling students were first generation college students, and most ethnic students were bilingual, fluent in English and their native languages. Participants had counselling experiences with clients from diverse ethnic backgrounds. On campus, the overall student population included American Indians (0.64 %), Blacks (5 %), Asians (15 %), Whites (33 %), and Latinos (36 %). In the local community, the population included American Indian and Alaska Native persons (2 %), Asians (9 %), Biracial/Multiracial persons (2 %), Blacks (6 %), Whites (35 %), and Latinos (49 %) (US Census Bureau 2010).

Participants received training in a person-centred approach that stresses clients’ problem-solving potentials, self-direction and self-exploration, and the use of genuineness, warmth, accurate empathy, respect, confrontation and a non-judgemental approach to build therapeutic relationships (Corey 2009). At the same time, participants learned counselling in multicultural contexts through taking Multicultural Counselling as a core course and other courses that reinforce a multicultural perspective throughout the programme.

Participants were drawn from students enrolled in the courses: Counselling Theories, Counselling Techniques, Individual Practicum, Advanced Marriage and Family Therapy Practicum, and Internship. These courses are taken in sequence, with an experiential component involving direct counselling experiences with increasing levels of complexities from Counselling Theories to Internship. Having participants from the above sequential courses generated a cross-sectional sample of counselling students at different phases of their professional training development.

The sample in this study consisted primarily of female students (*N* = 188, 81 %), with fewer male students (*N* = 45, 19 %). The race/ethnicity make-up of the sample was largely Latino (*N* = 92, 40 %) and White (*N* = 80, 34 %), followed by Asian/Pacific Islander (*N* = 17, 7 %), Biracial/Multiracial (*N* = 15, 6 %), African American (*N* = 11, 5 %), and Others (*N* = 18, 8 %). Due to the uncertainty of the ethnicity and small number of respondents in the ‘Others’ category, this study did not include this category in the analysis of ethnic differences. No participants were Native American. The ages of the students ranged from 19 to 61 with a mean of 27.52 and a standard deviation of 6.39. Due to incomplete data for all participants, some students were eliminated from subsequent analyses. The numbers varied by analysis but ranged between 68 and 88 participants.

### Procedure

The first author contacted instructors teaching the Counselling Theories, Counselling Techniques, Individual Practicum, Advanced Marriage and Family Therapy Practicum, and Internship courses to get approval to visit their class meetings. The same researcher then visited each class at the end of the semester to invite students to participate voluntarily in this study. Participants were asked to provide demographic information and give responses to the instrument used in this study. Consent forms with disclosures about confidentiality and potential benefits and harms were given to students before their participation. Participants were assured that they could withdraw from participation at any time.

### Instrument

The instrument used to measure counselling self-efficacy was the Counselling Self-Estimate Inventory (COSE) designed by Larson, *et al.* (1992). COSE consists of 37 statements with 6-point Likert-type responses on a scale of 1 (strongly disagree) to 6 (strongly agree). Higher total scores denote stronger perceptions of one’s counselling self-efficacy. The factor analysis completed by Larson, *et al.* identified five dimensions or subscales underlying counselling self-efficacy: “confidence in executing microskills, attending to process, dealing with difficult client behaviours, behaving in a culturally competent way, and being aware of one’s values” (p. 117). An internal consistency reliability of α = .93 for the total score and research support for validity can be found in Larson, *et al.* (1992). The skills being surveyed in the instrument are congruent with the skills emphasized in the counsellor education programme involved in this study.

The internal consistency reliability coefficients for the five factors of Micro Skills, Counselling Process, Difficult Client Behaviours, Cultural Competence, Awareness of Values and Total Self-Efficacy appear in Table 1. Cronbach’s Alpha reliabilities range from .40 to .93. The alpha for the Awareness of Values subscale was low, at .40. The range was much higher at .71 to .93 without the Awareness of Values subscale included. Coefficients of .70 or higher are considered acceptable (Groth-Marnat 1999). Internal consistency for Awareness of Values was low, which may indicate that this subscale was measuring more than one construct. However, the internal consistency reliability for these data was healthy, at .93 for Total Self-Efficacy.

**Table 1:** Reliabilities for Counselling Self-Efficacy—Total and Subscales

Variables	Cronbach’s Alpha
Self-Efficacy Total	.93
Micro Skills	.88
Counselling Process	.85
Difficult Client Behaviours	.78
Cultural Competence	.71
Awareness of Values	.40

## Analysis

Three series of one-way ANOVAs were run for the dependent variables of Total Self-Efficacy, Micro Skills, Difficult Client Behaviour, Counselling Procedure, Cultural Competence and Awareness of Values for each of the independent variables. The first series tested each dependent variable by Age Groups (19–22, 23–29, 30–39 and 40+). The second series tested each dependent variable by Gender (Male and Female). The third series tested each dependent variable by Ethnicity (African American, Asian, Latino, White and Biracial/Multiracial). No factorial ANOVAs with combinations of independent variables were run due to insufficient participants to fill all cells, so no interactions were tested.

## Results

The means, standard deviations, sample sizes and ANOVA results for Total Self-Efficacy and subscales by age appear in Table 2, by gender in Table 3, and by ethnicity in Table 4. Post hoc tests for significant results are reported in Table 5. Some of the post hoc results may appear counter-intuitive, as some pairs of means with smaller mean differences are significant at times, when other pairs with larger differences are not. This is because significance is in part a function of sample sizes, and all groups in this study did not have the same numbers of participants.

**Table 2:** Means, standard deviations, sample sizes and ANOVA results for Counselling Self-Efficacy—Total and Subscales by age groups

Variable	*M*	SD	*N*	*F*	*df*	*p*	Eta Squared
Total Self-Efficacy							
19–22 years	163.48	18.46	25	2.45	3,141	.07	.05
23–29	170.43	21.49	80				
30–39	177.07	20.24	30				
40+	162.30	21.50	10				
Total	170.04	21.06	145				
Micro Skills							
19–22 years	57.12	6.22	25	.77	3,151	.52	.02
23–29	56.88	7.01	89				
30–39	58.97	6.41	31				
40+	57.10	5.65	10				
Total	57.35	6.68	155				
Difficult Client Behaviours							
19–22 years	27.04	4.61	25	1.79	3,159	.15	.03
23–29	29.18	5.41	96				
30–39	29.94	6.22	32				
40+	27.00	6.45	10				
Total	28.87	5.57	163				
Counselling Procedures							
19–22 years	42.28	6.89	25	2.39	3,157	.07	.04
23–29	43.90	7.64	94				
30–39	46.81	7.76	32				
40+	41.00	8.81	10				
Total	44.05	7.73	161				
Cultural Competence							
19–22 years	19.64	2.98	25	.61	3,160	.61	.01
23–29	20.32	2.85	97				
30–39	20.38	2.89	32				
40+	19.50	2.46	10				
Total	20.18	2.85	164				
Awareness of Values							
19–22 years	17.40	2.81	25	2.10	3,159	.10	.04
23–29	18.58	3.17	97				
30–39	19.35	2.98	31				
40+	17.70	3.16	10				
Total	18.50	3.12	163

**Table 3:** Means, standard deviations, sample sizes and ANOVA results for Counselling Self-Efficacy—Total and Subscales by gender

Variable	*M*	SD	*N*	*F*	*df*	*p*	Eta Squared
Total Self-Efficacy							
Male	170.24	25.00	34	.000	1,144	.995	<.001
Female	170.26	19.95	112				
Total	170.25	21.14	146				
Micro Skills							
Male	57.51	8.51	35	.008	1,154	.93	<.001
Female	57.40	6.15	121				
Total	57.42	6.72	156				
Difficult Client Behaviours							
Male	29.59	5.62	37	.72	1,162	.40	.004
Female	28.71	5.57	127				
Total	28.91	5.58	164				
Counselling Procedures							
Male	43.72	9.03	36	.12	1,160	.73	.001
Female	44.22	7.37	126				
Total	44.11	7.74	162				
Cultural Competence							
Male	19.86	3.19	37	.55	1,163	.46	.003
Female	20.26	2.74	128				
Total	20.17	2.84	165				
Awareness of Values							
Male	18.50	2.97	36	.003	1,162	.96	<.001
Female	18.53	3.18	128				
Total	18.52	3.13	164

**Table 4:** Means, standard deviations, sample sizes and ANOVA results for Counselling Self-Efficacy—Total and Subscales by ethnicity

Variable	*M*	SD	*N*	*F*	*df*	*p*	Eta Squared
Total Self-Efficacy							
Afr. Amer.	181.50	6.66	6	4.22	4,141	.003	.11
Asian	163.50	19.91	16				
Latino	176.02	24.27	57				
White	163.80	17.01	59				
Biracial	181.88	16.73	8				
Total	170.25	21.14	146				
Micro Skills							
Afr. Amer.	61.00	5.18	6	2.36	4,151	.06	.06
Asian	56.00	6.57	16				
Latino	58.14	7.82	63				
White	56.10	5.41	62				
Biracial	61.67	5.41	9				
Total	57.42	6.72	156				
Difficult Client Behaviours							
Afr. Amer.	31.33	4.59	6	2.54	4,159	.04	.06
Asian	28.38	5.57	16				
Latino	30.16	6.19	68				
White	27.38	4.79	65				
Biracial	29.78	4.41	9				
Total	28.90	5.58	164				
Counselling Procedures							
Afr. Amer.	47.17	5.67	6	2.35	4,157	.06	.06
Asian	42.44	6.46	16				
Latino	45.50	8.58	68				
White	42.27	7.01	63				
Biracial	47.44	6.50	9				
Total	44.11	7.74	162				
Cultural Competence							
Afr. Amer.	22.33	2.07	6	6.82	4,160	<.001	.15
Asian	20.06	3.04	16				
Latino	21.03	2.59	70				
White	18.92	2.75	65				
Biracial	21.38	1.77	8				
Total	20.17	2.84	165				
Awareness of Values							
Afr. Amer.	19.67	3.50	6	2.22	4,159	.07	.05
Asian	16.63	3.10	16				
Latino	18.83	3.29	69				
White	18.41	2.92	64				
Biracial	19.67	2.00	9				
Total	18.52	3.13	164

**Table 5:** Significant Post-Hoc Results

Independent Variables	Dependent Variables	Larger and Smaller Group Means	*p*
Ethnicity	Total Self-Efficacy	African Amer.(*M* = 181.50) > White (*M* = 163.80)	.04
		Latino (*M* = 176.02) > Asian (*M* = 163.50)	.03
		Latino (*M* = 176.02) > White (*M* = 163.80)	.001
		Biracial (*M* = 181.88) > Asian (*M* = 163.50)	.04
		Biracial (*M* = 181.88) > White (*M* = 163.80)	.02
Ethnicity	Difficult Client Behaviours	Latino (*M* = 30.16) > White (*M* = 27.38)	.004
Ethnicity	Cultural Competence	African Amer. (*M* = 22.33) > White (*M* = 18.92)	.003
		Latino (*M* = 21.03) > White (*M* = 18.92)	<.001
		Biracial (*M* = 21.38) > White (*M* = 18.92)	.02

In examining the ANOVA results, there were no significant differences between age groups for Total Self-Efficacy or for any of the subscales. However, the results approached significance for two variables: Total Self-Efficacy and Counselling Procedures. For Total Self-Efficacy (*F* = 2.45, *df* = 3, 141, *p* = .07, *eta* ^*2*^ = .05), the 30 to 39 year olds had the highest mean (*M* = 177.07). This was followed by the mean for 23 to 29 year olds (*M* = 170.43), which was greater than the mean for 19 to 22 year old students (*M* = 163.48) or for 40+ students (*M* = 162.30). In addition, the age findings approached significance for Counselling Procedures (*F* = 2.39, *df* = 3, 157, *p* = .07, *eta* ^*2*^ = .04). The means ranked from highest to lowest were for 30 to 39 year olds (*M* = 46.81), 23 to 29 year olds (*M* = 43.90), 19 to 22 year olds (*M* = 42.28), and finally for 40+ students (*M* = 41.00).

None of the ANOVAs for Total Self-Efficacy or for any of the subscales was significant for gender. In fact, all differences between male and female means were less than 1 point for all dependent variables, indicating a strong degree of similarity between males and females.

All of the ethnicity ANOVA results were either significant or approached significance. Significant differences on ethnicity were found for Total Self-Efficacy, Difficult Client Behaviours, and Cultural Competence, and the results for Micro Skills, Counselling Procedures and Awareness of Values approached significance. Ethnicity differences were found for Total Self-Efficacy (*F* = 4.22, *df* = 4, 141, *p* = .003, *eta* ^*2*^ = .11) with 11 % of the variance accounted for. Biracial (*M* = 181.88) and African-American persons (*M* = 181.50) were nearly tied for the highest scores. They were followed by Latino students (*M* = 176.02), while White (*M* = 163.80) and Asian students (*M* = 163.50) were nearly tied at the low end.

Post hoc tests revealed that on Total Self-Efficacy, African Americans had significantly higher means than White students (*p* = .04), Latinos were higher than Asians (*p* = .03) and Whites (*p* = .001), and Biracial students were significantly higher than both Asians (*p* = .04) and Whites (*p* = .02). Significant main effects for ethnicity were found for the Difficult Client Behaviours subscale (*F* = 2.54, *df* = 4, 159, *p* = .04, *eta* ^*2*^ = .06), with 6 % of the variance accounted for. The ordering of means from highest to lowest were as follows: African American (*M* = 31.33), Latino (*M* = 30.16), Biracial (*M* = 29.78), Asian (*M* = 28.38) and White students (*M* = 27.38). Post hoc tests found only Latino students significantly higher for Difficult Client Behaviours than White students (*p* = .004). Significant ethnic differences were found in Cultural Competency (*F* = 6.82, *df* = 4, 160, *p* < .001, *eta* ^*2*^ = .15), with 15 % of the variance accounted for. The ordering of means from highest to lowest were as follows: African American (*M* = 22.33), Biracial (*M* = 21.38), Latino (*M* = 21.03), Asian (*M* = 20.06), and White students (*M* = 18.92). The post hoc tests revealed that African American (*p* = .003), Latino (*p* = <.001), and Biracial students (*p* = .02) were all significantly higher than White students.

Results approached significance for ethnic differences on Micro Skills (*F* = 2.36, *df* = 4, 151, *p* = .06, *eta* ^*2*^ = .06), with Biracial (*M* = 61.67) and African American students (*M* = 61.00) having similar high scores, followed by Latino (*M* = 58.14), and with White (*M* = 56.10) and Asian (*M* = 56.00) students having similar low scores. Results approached significance for ethnic differences on Counselling Procedures (*F* = 2.35, *df* = 4, 157, *p* = .06, *eta* ^*2*^ = .06), with Biracial (*M* = 47.44) and African American students (*M* = 47.17) having similar high scores, followed again by Latino (*M* = 45.50), and with Asian (*M* = 42.44) and White students (*M* = 42.27) having similar low scores.

Finally, results also approached significance for ethnic differences on Awareness of Values (*F* = 2.22, *df* = 4, 159, *p* = .07, *eta* ^*2*^ = .05), with African American and Biracial students (*M* = 19.67) both having the same high means, closely followed by Latinos (*M* = 18.83) and Whites (*M* = 18.41), and all scoring higher than Asian students (*M* = 16.63).

## Discussion

Differences in Total Self-Efficacy and Counselling Procedures among age groups approached significance, showing that 30 to 39 year olds ranked highest in these two variables when compared to 40+ year olds and 19 to 22 year olds. From a generational perspective, a likely stronger sense of individualism and autonomy may give Generation Xers (30–50 years old) an edge in developing greater counselling self-efficacy than the Generation Yers (10–30 years old). From Erikson’s psychosocial developmental perspective during middle adulthood, 30–39 year olds are concerned typically more about creative and meaningful work while 19–22 year olds are still tackling the task of forming intimate relationships as young adults (Evans *et al.* 2010). These factors may render the 30–39 year olds more self-efficacious in their professional abilities. Although the 40+ year olds belong broadly to the same generation and developmental stage as the 30–39 year olds, they showed less counselling self-efficacy. Since 40+ year olds most likely enter the counsellor education programme with more years of working experiences in another career and potentially a shorter remaining work span than 30–39 years old, they may have higher expectations for doing well, greater sensitivity to making mistakes, a stronger tendency to self-scrutinize and consequently lower counselling self-efficacy.

No significant difference on counselling self-efficacy based on gender was found in this study. This supports the observation that male college students showed consistent self-efficacy in both traditionally female and male occupations (Betz and Hackett 1981), but contradicts the concerns over Gender Role Conflict (GRC) suggested by Wester and Vogel (2002). This may indicate that males who pursue counselling as a profession typically possess personality traits that match the counselling profession, and therefore, are not necessarily socialized into traditional male roles. Despite these findings, further research is needed to explore the association between counselling self-efficacy and the gender role orientation and sex of the counsellor.

Regarding differences between ethnicities on counselling self-efficacy, Biracial participants and African Americans reported the highest means on Total Self-Efficacy, followed by Latinos, Whites and Asians. Post Hoc results showed significant group differences with African Americans having higher means than White students, while Latinos were higher than Asians and Whites. In a profession dominated by Whites, White students in this counsellor education programme did not have higher counselling self-efficacy than ethnic minorities in mastering skills designed by theories embedded in Western culture. It should be noted that White participants in this study were not the majority group in their programme (30 % White; 52 % Latino), in the university (33 % White; 36 % Latino), or in the community (35 % White; 49 % Latino). The trend of diversifying ethnicity of faculty, counselling student population, and counselling clientele, together with the push for multiculturalism, are factors for further consideration.

On the subscale of Difficult Client Behaviour, ethnic minority participants (i.e., Latino, African American, Bi-Racial, and Asian) reported higher levels of counselling self-efficacy than Whites, with Latino students significantly higher than Whites. Difficult clients are described as noncommittal, indecisive, unmotivated, or inexpressive in the instrument utilised (COSE). Berger and Morrison (1984) contend that minority counsellors hold more positive perceptions of successful treatment with difficult clients than their White counterparts due to cultural differences in expectations of what constitutes a “good” client. In our results, minority counsellors may have been more confident in their ability to form therapeutic alliances with difficult clients based on a non-Western cultural perspective, while White counsellors may lack confidence in effecting meaningful change with these types of clients. Minority counsellors may also be more cognizant of clients who question the efficacy of traditional forms of therapy. Indeed, racial and ethnic minorities in the U.S. reportedly lag behind their White counterparts in using mental health services (U.S. Department of Health and Human Services 2001).

On the subscale of Cultural Competence, African American, Latino, and Biracial students reported higher degrees of self-efficacy than Whites. This supports a study by Pope-Davis *et al.* (1995) that found a significant relationship existed between multicultural self-efficacy and ethnicity, with ethnic students showing a higher sense of competence than their White peers. According to Butler-Byrd (2010), White counsellor trainees “experience a great deal of disequilibrium, expressed as passive aggression, denial of differences, and/or of the significance of their own cultural/ethnic background and the backgrounds of others, or denial, guilt, and shame about their power and privilege” (p. 14). In addition, Parker and Schwarts (2002) suggest that White counsellors experience a sense of shame that may impede the advancement of multicultural competence. In the current study, lower levels of self-efficacy for Whites on this construct may also indicate an increased understanding and appreciation of the impact of institutional oppression on ethnic minorities while becoming aware of their own personal biases and societal prejudices.

While ethnic minorities demonstrated higher counselling self-efficacy than White students in this study, the Asian group consistently reported low counselling self-efficacy: lowest in Total Self-Efficacy, and second lowest in Difficult Client Behaviour and Cultural Competence. Many factors and different perspectives can be drawn on to understand this phenomenon. First, Asians such as Chinese may express lower degrees of self-efficacy than the level they truly believe due to cultural values for modesty (Yuen *et al.* 2004). Therefore, scores gathered from participants may not reflect their true sense of counselling self-efficacy. Also, Asian students may struggle with cultural conflicts between Western approaches to counselling and Asian styles of interpersonal relationships (Wohl 1982; Zane *et al.* 1991) and/or limited development in the sense of personal agency (Mutchler and Anderson 2010). Some Asian students may believe more in collective efficacy than individual self-efficacy (Kim and Omizo 2005).

Another factor to consider is that the Asian students in this counsellor education programme were predominantly Hmong students (50 %), along with a few being Indian, Chinese, Japanese and Korean. According to Duffy *et al.* (2004), the Hmong in the U.S. are members of an ethnic group from Laos and most resettled in the U.S. as refugees beginning about 50 years ago. Their cultural heritage can be traced back to China. While typically about half of Hmong are Christians, others uphold their beliefs in animism and the use of a Shaman to mediate between the worlds of spirits. Elders and Shamans are often sought out for guidance and healing. Having a relatively brief history in the U.S. and a belief in traditional healing practices are factors to consider in the development of self-efficacy among these students.

### Implications

Due to the limited scope of this study, confounding variables such as life experiences, auxiliary sources of training, and acculturation statuses that may influence trainees’ responses to the COSE were not controlled for. For example, students were not differentiated between those born in the U.S. and those being trained for U.S. contexts post-graduation. Despite such limitations, findings from this study render support to the potential impact that diversity factors may have on counselling students’ relative self-efficacy. Implications drawn from this study may heighten counsellor educators’ sensitivity to their students’ backgrounds during the training process.

While typically there are “more differences within a particular group than there are differences between groups” (Dana *et al.* 2008, p. 60), trainees’ age, gender, and ethnicity do play a part in their personal growth and identity development. Evolving self-appraisal, belief systems and interpersonal conduct that conflicts with the counselling behaviours prescribed by a programme may result in negative feedback from clients, peers, faculty, and ultimately the trainees themselves. It is imperative that counsellor educators develop training programmes that underscore the importance of matching training approaches with people’s sense of self and/or self-efficacious beliefs (Klassen 2004). Factors arising from trainees’ backgrounds that impact the development of counselling self-efficacy could be an integral part of on-going dialogues between trainees and their supervisors.

Although this study was conducted in the U.S., the findings may have implications for the training and development of professional counsellors at a global level. Along with the globalization of the counselling profession comes an indigenization process, which is taking place in different parts of the world (Leung *et al.* 2009). Indigenization involves the dual process of “transforming and adapting imported psychological theories, concepts, and methods” to apply to a local cultural context and developing “theories, categories, and constructs from within a culture, [and] using indigenous information…as primary sources of knowledge” (p. 116). No matter how homogeneous a society may appear, the counselling theories, concepts, and methods emerging from the process of balancing “cultural-universals” and “cultural specifics” (p. 117) may pose different challenges to counsellor trainees from different age, gender, and ethnicity groups, which may in turn impact trainees’ counselling self-efficacy.

The present study suggests that even White students, who are considered to possess privileges based on being the majority group in the U.S. and are trained in counselling theories, concepts, and methods most compatible with a Western cultural heritage, do not necessarily enjoy a higher counselling self-efficacy than counterparts from racial minority groups. Withholding the assumption that a particular approach in counselling is readily suitable for all counselling students to master may be helpful. Even in societies where a certain race/ethnicity seems to dominate the whole community, counsellor trainees with different diversity statuses may bring with them a wide range of life experiences, self-concepts, and worldviews that in turn impact their counselling self-efficacy. For example, a group of Chinese counsellor trainees in Mainland China may be as diverse as, if not more than, the group of counsellor trainees who participated in this study. Chinese of different age groups born between 1949 and 2011 would have been exposed to different ideologies, living conditions, family make-up, and values. The relative influence of traditional Chinese cultural systems, such as Confucianism, and western cultures differs from one generation to another. Differences in acculturation among ethnic groups in the U.S. may be present in non-U.S. countries through globalization. In any country, a continuum where people can fall at any point between holding traditional or modern values may exist. Gender role socialization may also differ from one generation to another, from rural to urban locations, from embracing indigenous values to western beliefs. Counsellor preparation programmes might benefit from a greater awareness of the interconnectedness between counselling approaches and trainees’ cultural orientations.

## Recommendations for Future Research

To deepen the understanding of the role that diversity factors play on counselling students’ sense of counselling efficacy, exploration of specific influences of diversity status on counsellor self-efficacy is warranted. Future studies that include international students, immigrants, and participants with differing levels of acculturation may be beneficial in explaining the relationship between alternative worldviews and counsellor trainees’ perceptions of self-efficacy. Interviews with counsellor trainees who have been advised of their scores for the COSE to explore how they interpret the results and explain their relative strength in counselling self-efficacy would also provide further insights into factors affecting counselling self-efficacy.

## References

[CR1] Bandura A (1995). Self-efficacy in changing societies.

[CR2] Beckenbach J, Patrick S, Sells J (2009). The evaluation of participants’ perception of session impact: do counsellors-in-training, volunteer clients, and extra-credit/class required clients view session impact differently?. British Journal of Guidance and Counselling.

[CR3] Berger A, Morrison TL (1984). Clinical judgments of easy vs. difficult clients by counsellor trainees. Journal of Clinical Psychology.

[CR4] Berry JM, West RL (1993). Cognitive self-efficacy in relation to personal mastery and goal setting across the life span. International Journal of Behavioral Development.

[CR5] Betz, N. E., & Hackett, G. (1981). The relationship of career-related self-efficacy expectations to perceived career options in college women and men. *Journal of Counselling Psychology, 28*(5), 399–410. Retrieved from http://web.ebscohost.com.hmlproxy.lib.csufresno.edu/ehost/pdfviewer/pdfviewer?vid=3&hid=126&sid=414c558c-4627-4159-88d1-768dc3c06bad%40sessionmgr115.

[CR6] Butler-Byrd NM (2010). An African American supervisor’s reflections on multicultural supervision. Training and Education in Professional Psychology.

[CR7] Carnevale, A. P., Strohl, J., & Melton, M. (2011). What’s it worth? The economic value of college majors. ACAeNews Volume XIII, No. 11. Retrieved from http://www.counseling.org/Publications/Newsletters.aspx.

[CR8] US Census Bureau (2010). State and county quickfacts: Fresno County, California. Retrieved from http://quickfacts.census.gov/qfd/states/06/06019.html.

[CR9] Chung RCY, Bemak F (2002). The relationship of culture and empathy in cross-cultural counselling. Journal of Counselling and Development.

[CR10] Corey G (2009). Theory and practice of counseling and psychotherapy.

[CR11] Council for Accreditation of Counselling & Related Educational Programs (CACREP) (2009). *2009 Standards.* Retrieved from http://www.cacrep.org/template/index.cfm.

[CR12] Cross WE (1991). Shades of black: Diversity in African American identity.

[CR13] Crutchfield LB, Baltimore ML, Felfeli M, Worth S (2000). Empathic responding skills across counselor education training tracks: a comparison study. Journal of Humanistic Counselling, Education and Development.

[CR14] Dana RH, Gamst GC, Der-Karabetian A (2008). CBMCS multicultural training program.

[CR15] Duffy J, Harmon R, Ranard DA, Thao B, Yang K (2004). The Hmong: An introduction to their history and culture.

[CR16] Evans NJ, Forney DS, Guido FM, Patton LD, Renn KA (2010). Student development in college: Theory, research, and practice.

[CR17] Ferdman BM, Gallegos PI, Wijeyesinghe CL, Jackson BW (2001). Racial identity development and Latinos in the United States. New perspectives on racial identity development: A theoretical and practical anthology.

[CR18] Gelso CJ (2010). The diversity status of the psychotherapist: editorial introduction. Psychotherapy: Theory, Research, Practice, Training.

[CR19] Gerstein LH, Heppner PP, Ægisdóttir S, Leung SMA, Norworthy KL, Gerstein LH, Heppner PP, Ægisdóttir S, Leung SMA, Norsworthy KL (2009). Cross-cultural counseling: History, challenges, and rationale. International handbook of cross-cultural counseling.

[CR20] Groth-Marnat G (1999). Handbook of psychological assessment.

[CR21] Helms JE (1993). Black and white racial identity: Theory, research and practice.

[CR22] Heppner PP, Ægisdóttir S, Leung SMA, Duan C, Helms JE, Gerstein LH, Pedersen PB, Gerstein LH, Heppner PP, Ægisdóttir S, Leung SMA, Norsworthy KL (2009). The intersection of multicultural and cross-national movements in the United States. International handbook of cross-cultural counseling.

[CR23] Horse PG, Wijeyesinghe CL, Jackson BW (2001). Reflections on American Indian identity. New perspectives on racial identity development: A theoretical and practical anthology.

[CR24] Jaafar, W. M. W., Mohamed, O., Bakar, A. R., & Tarmizi, R. A. (2009). The influence of counselling self-efficacy towards trainee counsellor performance. *International Journal of Learning, 16*(8), http://www.Learning-Journal.com, ISSN 1447–9494.

[CR25] Kim J, Wijeyesinghe CL, Jackson BW (2001). Asian American identity development theory. New perspectives on racial identity development: A theoretical and practical anthology.

[CR26] Kim BSK, Omizo MM (2005). Asian and European American cultural values, collective self-esteem, acculturative stress, cognitive flexibility, and general self-efficacy among Asian American college students. Journal of Counselling Psychology.

[CR27] Klassen RM (2004). Optimism and realism: a review of self-efficacy from a cross-cultural perspective. International Journal of Psychology.

[CR28] LaFromboise T, Coleman HLK, Gerton J (1993). Psychological impact of biculturalism: evidence and theory. Psychological Bulletin.

[CR29] Larson LM (1998). The social cognitive model of counsellor training. The Counselling Psychologist.

[CR30] Larson LM, Suzuki LA, Gillespie KN, Potenza MT, Bechtel MA, Toulouse AL (1992). Development and validation of the Counselling Self-Estimate Inventory. Journal of Counselling Psychology.

[CR31] Lent RW, Hoffman MA, Hill CE, Treistman D, Mount M, Singley D (2006). Client-specific counsellor self-efficacy in novice counsellors: relation to perceptions of session quality. Journal of Counselling Psychology.

[CR32] Leung SMA, Clawson T, Norsworthy KL, Tena A, Szilagyi A, Rogers J, Gerstein LH, Heppner PP, Ægisdóttir S, Leung SMA, Norsworthy KL (2009). Internationalization of the counselling profession: An indigenous perspective. International handbook of cross-cultural counseling.

[CR33] Marquié JC, Huet N (2000). Age differences in feeling-of-knowing and confidence judgment as a function of knowledge domain. Psychology and Aging.

[CR34] Mutchler, M., & Anderson, S. (2010). Therapist personal agency: A model for examining the training context. *Journal of Marital and Family Therapy, 36*(4), 511–525. doi:10.1111/j.1752-0606.2010.00198.x. 10.1111/j.1752-0606.2010.00198.x 21039662

[CR35] Parker WM, Schwarts RC (2002). On the experience of shame in multicultural counselling: implications for white training-in-training. British Journal of Guidance and Counselling.

[CR36] Pearson QM (1999). Integrative empathy: training counsellors to listen with a theoretical ear. Journal of Humanistic Counselling, Education and Development.

[CR37] Pope-Davis DB, Reynolds AL, Dings JG, Ottavi (1994). Multicultural competencies of doctoral interns at university counseling centers: an exploratory investigation. Professional Psychology: Research and Practice.

[CR38] Pope-Davis DB, Reynolds AL, Dings JG, Nielson D (1995). Examining multicultural counseling competencies of graduate students in psychology. Professional Psychology: Research and Practice.

[CR39] Renn KA (2003). Understanding the identities of mixed race college students through a developmental ecology lens. Journal of College Development.

[CR40] Riemersma, M. (2010). The typical California MFT: 2010 CAMFT member practice and demographic survey. *The Therapist*, July/August, 1–7.

[CR41] Rogers CR (2007). The necessary and sufficient conditions of therapeutic personality change. Psychotherapy: Theory, Research, Practice, Training.

[CR42] Rowe W, Bennett SK, Atkinson DR (1994). White racial identity models: a critique and alternative proposal. Counselling Psychologist.

[CR43] Shallcross, L. (2009). From generation to generation: Working effectively with baby boomers, gen xers and millennials means undergoing a multicultural education. *Counseling Today Online*. Retrieved on March 25, 2010 from http://www.counseling.org/Publications/CounselingTodayArticles.aspx?Aguid=9e80a3ec-7ac9-419b-beab-d957ca3fa64a.

[CR44] U.S. Department of Health and Human Services (2001). Mental health: Culture, race, and ethnicity: A supplement to mental health: A report of the surgeon general – Executive summary.

[CR45] Wester SR, Vogel DL (2002). Working with the masculine mystique: male gender role conflict, counselling self-efficacy, and the training of male psychologists. Professional Psychology: Research and Practice.

[CR46] Wintersteen MB, Mensinger JL, Diamond GS (2005). Do gender and racial differences between patient and therapist affect therapeutic alliance and treatment retention in adolescents?. Professional Psychology: Research and Practice.

[CR47] Wohl J (1982). Eclecticism and Asian counselling: critique and application. International Journal for the Advancement of Counselling.

[CR48] Yuen MT, Chan R, Lau P, Lam MP, Shek DTL (2004). The counselling self-estimate inventory (COSE): does it work in Chinese counsellors?. Counselling Psychology Quarterly.

[CR49] Zane NWS, Sue S, Hu LT, Kwon JH (1991). Asian-American assertion: a social learning analysis of cultural difference. Journal of Counselling Psychology.

